# Alteration in Nuclear Factor-KappaB Pathway and Functionality of Estrogen via Receptors Promote Neuroinflammation in Frontal Cortex after 1-Methyl-4-Phenyl-1,2,3,6-Tetrahydropyridine Treatment

**DOI:** 10.1038/srep13949

**Published:** 2015-09-14

**Authors:** Soham Mitra, Nabanita Ghosh, Priyobrata Sinha, Nilkanta Chakrabarti, Arindam Bhattacharyya

**Affiliations:** 1 Immunology Lab, Department of Zoology, University of Calcutta, 35, Ballygunge Circular Road. Kolkata-700019, India; 2 Department of Physiology, University of Calcutta, 92, Acharya Prafulla Chandra Road, Kolkata-700009, India

## Abstract

The MPTP mediated neurodegeneration in substantia nigra has been well studied, but not the status of frontal cortex. The novelty of the present study is to explore the sex difference of frontal cortex during MPTP intoxication and to investigate the role of estrogen and its receptors in presence of glial cells in a time chase experiment; to identify which pathway of NF-kappaB exist to proceed the neuroinflammation; to investigate the estrogen binding with its nuclear or cytosolic receptors and whether any direct relation exists between estrogen receptor (ER) -beta and NF-kappaB molecules p65 and RelB. The progression of neurodegeneration occurred with the association of glial cells and functional (via its nuclear and cytosolic receptors) estrogen level. Both the canonical and/or non canonical pathways of NF-kappaB exist in frontal cortex of both the sexes after MPTP treatment. The homodimeric or heterodimeric form of ER-beta binds with NF-kappaB molecules p65 and RelB differently, but the canonical or non canonical pathways of NF-kappaB molecules could not be stopped or may be promoted. The changes in the molecular and cellular pattern in frontal cortex of both sexes during MPTP intoxication depends on the estrogen function via its nuclear or cytosolic estrogen receptors.

There are many environmental toxins that take part in introducing the mouse model of Parkinson’s disease (PD) with alternative pathological features. Among them, 1-methyl-4-phenyl-1,2,3,6-tetrahydropyridine (MPTP) appears to target the dopaminergic neurons and introduce itself as the most established animal models of idiopathic PD. The investigations made with MPTP induced mouse model of PD appears to reflect many important mechanistic pathways related to the disease initiation^1^,^2^. Typically, PD is characterized by decreased dopamine levels in the striatum and a loss of pigmented dopamine neuronal cells in the substantia nigra pars compacta and the similar features also exhibited by MPTP in a mouse model of PD^3^. Not only substantia nigra or striatum but cortical regions is also affected during the neurodegeneration in MPTP induced animal models of PD^4^,^5^.

Identifying environmental factors that predispose to the development of idiopathic PD via oxidative stress, mitochondrial dysfunction, Lewy pathology has proved elusive^6^,^7^. The major secondary cause of the disease that prolongs the disease progression is the neuroinflammation. Both epidemiological and genetic studies support a role of neuroinflammation in the pathophysiology of PD. Furthermore, post mortem studies confirm the involvement of innate as well as adaptive immunity and association of microglial/astroglial cells in the affected brain regions in patients with PD^8^,^9^. The MPTP treated animal model of PD also exhibits the neuroinflammation by releasing proinflammatory cytokines from microglial cells/astroglial cells in different time frame during the progression of the disease^10^,^11^. There are few an evidences present that not only substantia nigra, but cortical regions are also affected by neuroinflammation during the MPTP induced mouse model of PD^12^,^13^. However the exact role of glial cells during the progression of the neuroinflammation in PD is still remains elusive.

Increasing evidence suggests that estrogens protect the nigrostriatal dopaminergic pathway affected in PD. Animal studies also show that estrogens influence the synthesis, release, and metabolism of dopamine and can modulate dopamine receptor expression and function^14^. The neuroprotective action of estrogen depends on its receptor functionality and cellular localization when estrogen binds with the receptors α or β (ERα and ERβ) and that function of estrogen also exhibit anti inflammatory effect during PD^15^. The estrogen mediated protection also has effects on cortical areas during neuroinflammatory condition^16^.

Therefore, two opposite phenomena are present there during the progression of the disease- neuroinflammation and neuroprotection (by estrogen). Now the question arises- in spite of presence of estrogen how neuroinflammation proceeds to degenerate neurons in PD. To find the possibilities of the phenomenon, we have chosen the major transcription factor Nuclear factor κB (NF-κB) that are expressed in a wide variety of cells and tissues, including microglia, astrocytes, and neurons during the neuroinflammation. The NF-κB functions via two pathways- classical or canonical and alternative or non-canonical. Activation of the NF-κB pathway is mediated through the activity of the IKK kinase complex, targeting to alter NF-κB has been proposed as an approach to the treatment of acute and chronic inflammatory conditions, and the use of inhibitors specific for either IKKβ or IKKγ has now been found to inhibit neurodegeneration of dopaminergic neurons in murine and primate models of PD^17^. There is no clear evidence present, whether estrogen has any significant effect on modulation of NF-κB during the progression of the disease in cortical regions (mainly in frontal cortex; because motor imbalance and deterioration during the PD related to this region).

The present study has been designed to understand the possible crosstalk between estrogen action and NF-κB during the progression of the neuroinflammation/neurodegeneration in frontal cortex of the MPTP mediated mouse model of PD. The crosstalk has also been evaluated with respect to the status of microglial and astroglial cells and also in a time dependent manner.

## Results

### MPTP treatment decreased the expression level of FOX3 and FOX3 positive neuronal cell number in frontal cortex of both the sexes

**Figure 1:**
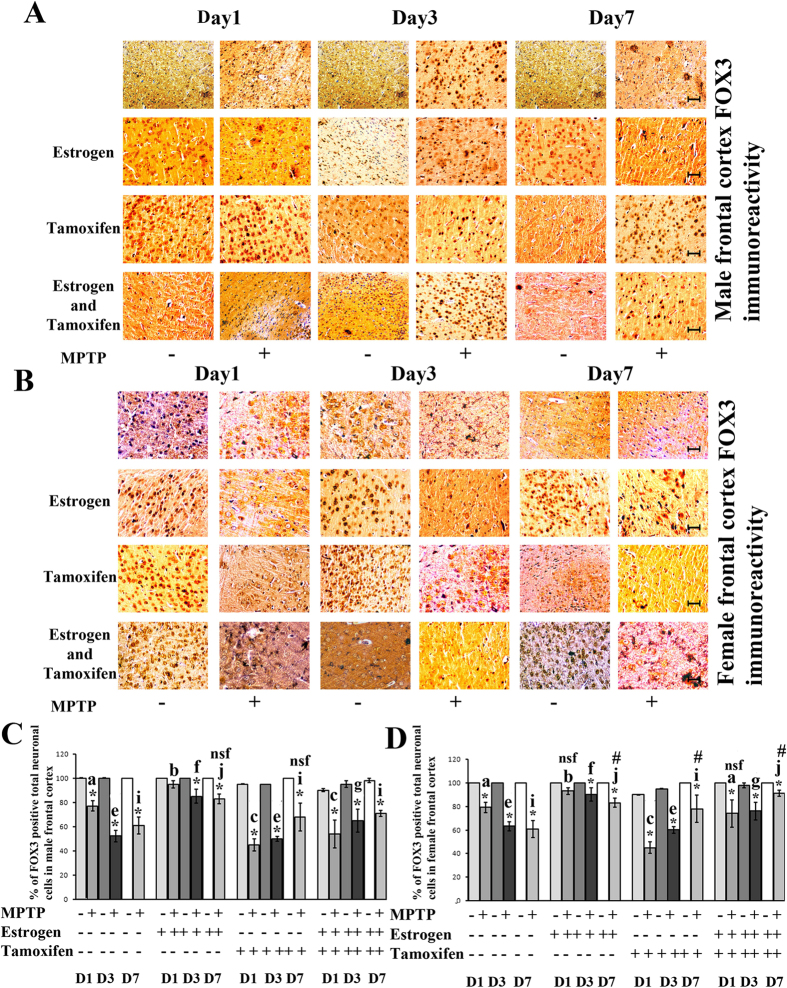
Changes in FOX3 positive neuronal population and immunoreactivity of FOX3 in frontal cortex of male and female mice brain at three different days of sacrifice in MPTP treatment and also estrogen and tamoxifen supplemented groups. Immunohistochemical analysis revealed that differential FOX3 immunoreactivity was found in MPTP and supplemented with estrogen and tamoxifen treated male frontal cortex (**A**) and female frontal cortex (**B**). Three different days of sacrifice are indicated by Day1, Day3 and Day7. Four different treatment groups with legitimate controls comprising MPTP, Estrogen supplemented MPTP, Tamoxifen supplemented MPTP and Estrogen + Tamoxifen supplemented MPTP represented by −/+ sign. Magnification of IHC images is 40×, and the scale bars = 40 μm. FACS analysis revealed the changes in FOX3 positive neuronal cell percentage due to MPTP treatment and also supplemented with estrogen and tamoxifen, represented as bar graphs in different shades in three different days of sacrifice of male and female frontal cortex compared to that of control (**C**,**D**). Three different days of sacrifice indicated by D1, D3, D7 represent day1, day3 and day7. Four different treatment groups with legitimate controls comprising MPTP, Estrogen supplemented MPTP, Tamoxifen supplemented MPTP and Estrogen + Tamoxifen supplemented MPTP represented by −/+ sign. Data are presented as mean ± SEM. (n = 5). Asterisks (*) indicate significant differences (p < 0.05, Student’s t-test) in values for different doses compared to respective controls. The same letter within a, b, c, d; e, f, g, h; i, j, k, l indicates no significant differences and different letters indicate significant differences (p < 0.05, ANOVA) between different treatment groups at the same day of sacrifice in male and female mice brain frontal cortex during treatment condition. Hash sign (#), nsf and dollar sign ($) indicates non significant value with different letters in between treated condition in different groups sacrificed at the same day in male and female mice group (**A**,**B**).

The results indicated that the FOX3 positive immunoreactivity and the neuronal population decreased significantly compared to the respective controls in frontal cortex of both the sexes during MPTP treatment (Fig. 1A–D) and decreased during the initial (day1) and the middle phase (day3) but did not recover at the later stage (day7) in frontal cortex of both the sexes after MPTP treatment (Fig. 1A–B). However, neuronal cells decreased more in progressive days of sacrifice after MPTP treatment (Fig. 1C,D). Estrogen supplement functioned as neuroprotective for MPTP treatment in both the sexes (Fig. 1C,D) but when estrogen receptors are blocked by tamoxifen, neurodegeneration became more deteriorated, that estrogen supplementation could not decrease the level of neurodegeneration (Fig. 1C,D). The pattern of these FOX3 positive neuronal changes did not alter at male and female frontal cortex during MPTP treatment, but the level of FOX3 positive neurodegeneration did vary among the male and female frontal cortex (Fig. 1C,D) depending upon the status of estrogen and its receptors.

### MPTP treatment differentially change the expression level of GFAP (glial fibrilary acidic protein) and GFAP positive astroglial cell number in frontal cortex of both the sexes

**Figure 2:**
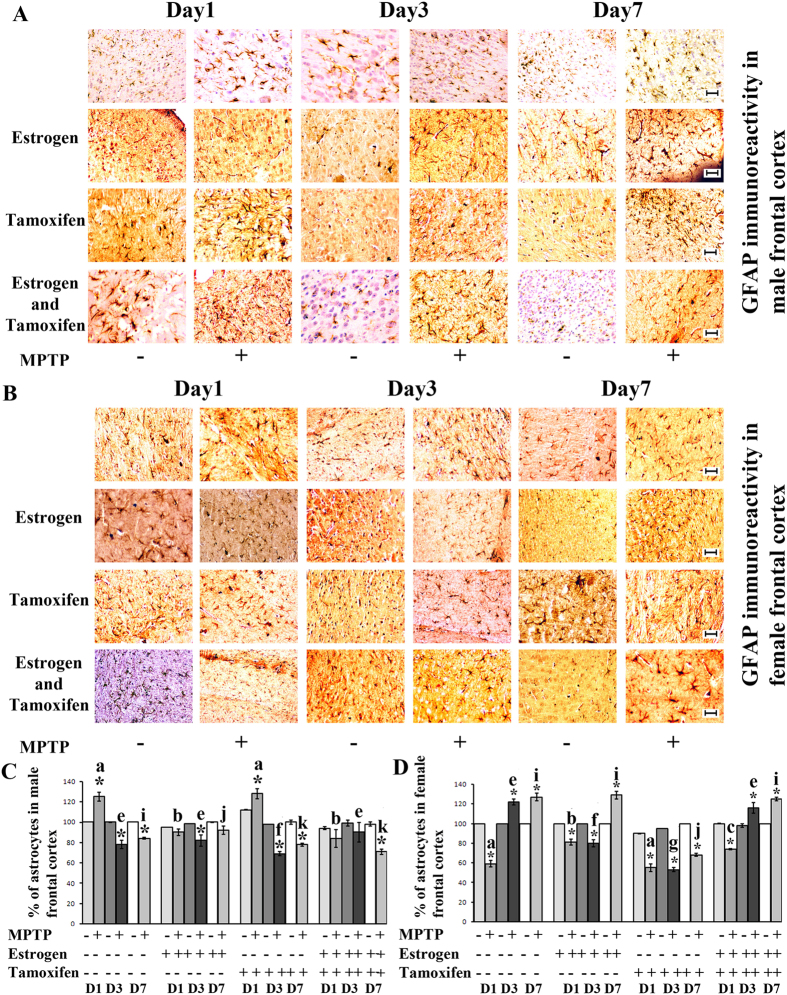
Changes in GFAP positive astrocytic population and immunoreactivity of GFAP in frontal cortex of male and female mouse brain at three different days of sacrifice in MPTP treatment and also estrogen and tamoxifen supplemented groups. Immunohistochemical analysis revealed that differential GFAP immunoreactivity was found in MPTP and supplemented with estrogen and tamoxifen treated male frontal cortex (**A**) and female frontal cortex (**B**). Three different days of sacrifice are indicated by Day1, Day3 and Day7. Four different treatment groups with legitimate controls comprising MPTP, Estrogen supplemented MPTP, Tamoxifen supplemented MPTP and Estrogen + Tamoxifen supplemented MPTP represented by −/+ sign. Magnification of IHC images is 40×, and the scale bars = 40 μm. FACS analysis revealed the changes in GFAP positive astrocytic cell percentage due to MPTP treatment and also supplemented with estrogen and tamoxifen, represented as bar graphs in different shades in three different days of sacrifice of male and female frontal cortex compared to that of control (**C**,**D**). Three different days of sacrifice indicated by D1, D3, D7 represent day1, day3 and day7. Four different treatment groups with legitimate controls comprising MPTP, Estrogen supplemented MPTP, Tamoxifen supplemented MPTP and Estrogen + Tamoxifen supplemented MPTP represented by −/+ sign. Data are presented as mean ± SEM. (n = 5). Asterisks (*) indicate significant differences (p < 0.05, Student’s t-test) in values for different doses compared to respective controls. The same letter within a, b, c, d; e, f, g, h; i, j, k, l indicates no significant differences and different letters indicate significant differences (p < 0.05, ANOVA) between different treatment groups at the same day of sacrifice in male and female mice brain frontal cortex during treatment condition. Hash sign (#), nsf and dollar sign ($) indicates non significant value with different letters in between treated condition in different groups sacrificed at the same day in male and female mice group (**A**,**B**).

In the present study, GFAP immunoreactivity, was the least on day3 and day7, increased on day1 of sacrifice in MPTP treated male (Fig. 2A,B) but decreased on day1 and increased on day3 and day7 of sacrifice in the MPTP treated female frontal cortex (2C and 2D). However the GFAP immunoreactivity did not change in estrogen supplemented MPTP treated group at on all three days of male and decreased initially, but increased in the middle and late phase of sacrifice in female frontal cortex. In tamoxifen supplemented MPTP group, the immunoreactivity increased at day1 and day7 of male and decreased on all the three days of sacrifice in female frontal cortex (Fig. 2A,B). Therefore the results depict one clear view is that GFAP immunoreactivity increased mostly when neuroprotection was much needed and when estrogen could not function via its receptors.

The percentage of astroglial population increased on day1 and decreased on day3 and day7 of sacrifice in male, however that percentage pattern was reverse in female frontal cortex of MPTP treated groups compared to their respective control (Fig. 2C,D). The unchanged pattern of astrocyte population, compared to the control has been found in male in estrogen supplemented MPTP treated group (Fig. 2C) but the population decreased in the same group of female frontal cortex compared to the controls (Fig. 2D). However, in tamoxifen supplemented MPTP treated group, the astrocyte population were being decreased significantly in both sexes except day1 of sacrifice in male frontal cortex (Fig. 2C,D respectively). Therefore, when estrogen could not function via its receptors, astrocytes might provide initial resistance to neurodegeneration by increasing the population size but loner its percentage decreased in male frontal cortex during MPTP treatment (Fig. 2C). However, in the same scenario, astrocytes did not put any resistance to the neurodegeneration and its number decreased in all the stages of neurodegeneration in female frontal cortex (Fig. 2D).

### MPTP treatment differently changed the expression level of Iba1 and Iba1 positive microglial cell number in frontal cortex of both the sexes

**Figure 3:**
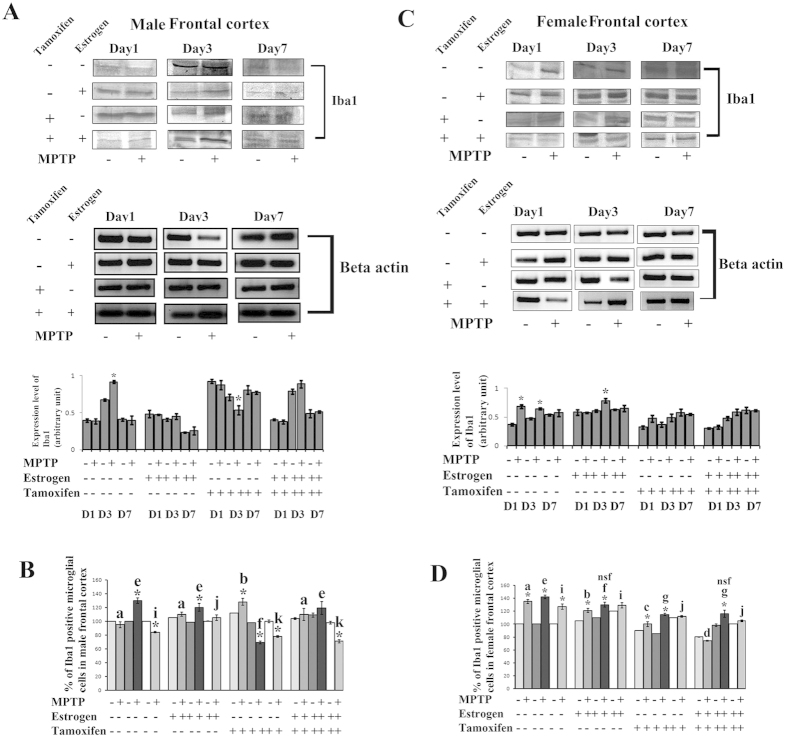
Changes in Iba1 positive microglial population and Iba1expression level in frontal cortex of male and female mice brain at three different days of sacrifice in MPTP treatment and also estrogen and tamoxifen supplemented groups. Analysis of western blots indicates that expression levels of Iba1 due to MPTP treatment and also supplemented with estrogen and tamoxifen. β-Actin was used as a reference control in both genders respectively (**A**,**C**). Data are presented as mean ± SEM. (n = 5). Three different days of sacrifice indicated by D1, D3, D7 represent day1, day3 and day7 in male and female frontal cortex. Four different treatment groups with legitimate controls comprising MPTP, Estrogen supplemented MPTP, Tamoxifen supplemented MPTP and Estrogen + Tamoxifen supplemented MPTP represented by −/+ sign in respective panels of both male and female frontal cortex (**A**,**C**). Densitometric analysis has been done by representative bar graphs. FACS analysis revealed the changes in Iba1 positive cell percentage due to MPTP treatment and also supplemented with estrogen and tamoxifen, represented as bar graphs in different shades in three different days of sacrifice of male and female frontal cortex compared to the control (**B,D**). Three different days of sacrifice indicated by D1, D3, D7 represent day1, day3 and day7. Four different treatment groups with controls comprising MPTP, Estrogen supplemented MPTP, Tamoxifen supplemented MPTP and Estrogen + Tamoxifen supplemented MPTP represented by −/+ sign. Data are presented as mean ± SEM. (n = 5). Asterisks (*) indicate significant differences (p < 0.05, Student’s t-test) in values for different doses compared to respective controls. The same letter within a, b, c, d; e, f, g, h; i, j, k, l indicates no significant differences and different letters indicate significant differences (p < 0.05, ANOVA) between different treatment groups at the same day of sacrifice in male and female mice frontal cortex during treatment. Hash sign (#), nsf and dollar sign ($) indicates non significant value with different letters in between treated condition in different groups sacrificed on the same day in male and female mice group (**B**,**D**).

In the MPTP treated group, the expression level of Iba1 increased significantly only in the middle phase (day3) of male (Fig. 3A) and initial (day1) and middle phase of female frontal cortex (Fig. 3C) compared to their respective controls. Estrogen and tamoxifen supplementation prior to MPTP treatment don’t have any significant effect on Iba1 expression level in both sexes (Fig. 3A,C respectively).

During MPTP treatment, the population size of Iba1 positive microglial cells increased significantly in all the three stages of sacrifice in MPTP treated female (Fig. 3B) but that percentage increased in middle and later phase (day7) of sacrifice; the cell number decreased significantly in treated male frontal cortex (Fig. 3D). It’s interesting to note that estrogen supplementation prior to MPTP treatment either increased the microglial cell percentage or kept the cell percentage unchanged in male and female frontal cortex (Fig. 3B,D) compared to respective controls. Further, more importantly, it has been found that when the estrogen receptors are blocked, the pattern change of microglial cells in female frontal cortex remained same in all the three days of sacrifice, but in males, the number of microglial cells decreased mostly except day1 of sacrifice (Fig. 3B).

### MPTP treatment differentially changes the level of TNF-α in frontal cortex of both the sexes

**Figure 4:**
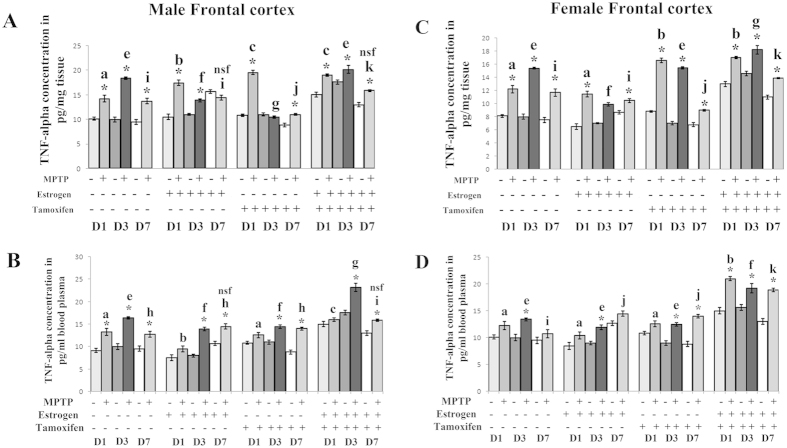
Changes of TNF-α level in frontal cortex and in blood plasma of male and female mice at three different days of sacrifice in MPTP treatment and also in estrogen and tamoxifen supplemented groups. ELISA analysis was done to evaluate the changes in TNF-α level due to MPTP treatment and also supplemented with estrogen and tamoxifen, represented as bar graphs in different shades in three different days of sacrifice in male and female frontal cortex (**A**,**C**), compared to that of respective control. ELISA analysis was also done to evaluate the changes in TNF-α level due to MPTP treatment and also supplemented with TNF-α and tamoxifen in male and female blood plasma, represented as bar graphs in different shades in three different days of sacrifice (**B,E**), compared to that of respective control. Three different days of sacrifice indicated by D1, D3, D7 represent day1, day3 and day7. Four different treatment groups with legitimate controls comprising MPTP, Estrogen supplemented MPTP, Tamoxifen supplemented MPTP and Estrogen + Tamoxifen supplemented MPTP represented by −/+ sign. Data are presented as mean ± SEM. (n = 5). Asterisks (*) indicate significant differences (p < 0.05, Student’s t-test) in values for different doses compared to respective controls. The same letter within a, b, c, d; e, f, g, h; i, j, k, l indicates no significant differences and different letters indicate significant differences (p < 0.05, ANOVA) between different treatment groups at the same day of sacrifice in male and female mice brain frontal cortex during treatment condition. Hash sign (#), nsf and dollar sign ($) indicates non significant value with different letters in between treated condition in different groups sacrificed at the same day in male and female mice group.

In the present study, TNF-α expression level increased significantly in all the three days of sacrifice in MPTP treated group with the highest level of expression in the middle phase of sacrifice in frontal cortex of both the sexes (Fig. 4A,C) as well as in the peripheral blood plasma (Fig. 4B,D). Previous evidences have shown that estrogen has anti-inflammatory function and also inhibit TNF-α secretion from microglial cells^18^. In the present study, estrogen supplement prior to the MPTP treatment has shown the sign of a decrease in elevated TNF-α level in frontal cortex of both the sexes (Fig. 4A,C). Interestingly, it has been found that tamoxifen supplement decreased the higher level of TNF-α in male frontal cortex except the initial stage of a sacrifice compared to the MPTP treated group (Fig. 4A). However, in female frontal cortex, the TNF-α level was increased more due to blocking of estrogen receptors (Fig. 4C).

### MPTP treatment differentially changes estrogen and aromatase level in frontal cortex of both the sexes

**Figure 5:**
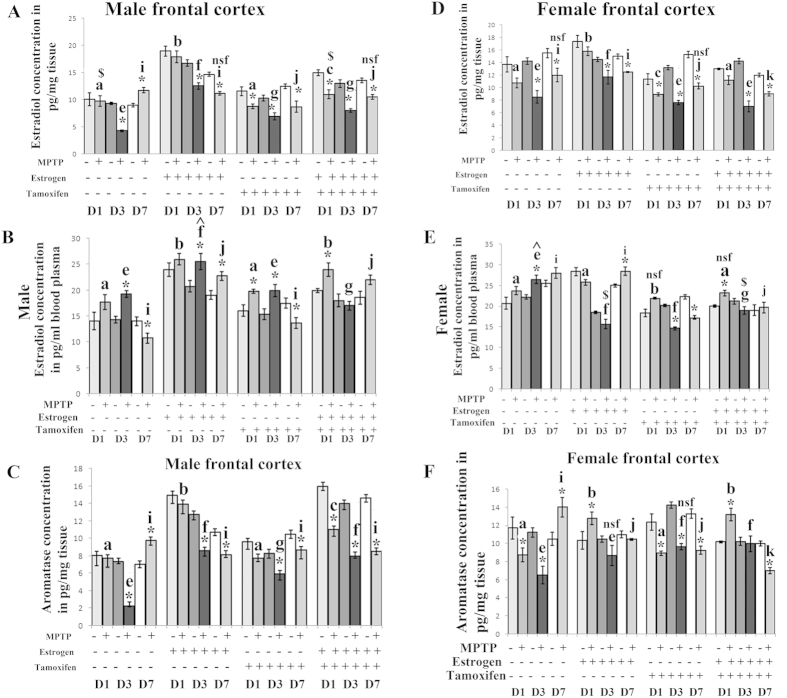
Changes of estrogen and aromatase level in frontal cortex and estrogen level in blood plasma of male and female mice at three different days of sacrifice in MPTP treatment and also estrogen and tamoxifen supplemented groups. ELISA analysis was done to evaluate the changes in estrogen level and aromatase level due to MPTP treatment and also supplemented with estrogen and tamoxifen in male and female frontal cortex (**A**,**D**,**C**,**F** respectively), represented as bar graphs in different shades in three different days of sacrifice, compared to that of respective control. ELISA analysis was also done to evaluate the changes in estrogen level due to MPTP treatment and also supplemented with estrogen and tamoxifen, represented as bar graphs in different shades in three different days of sacrifice in male and female blood plasma (**B,E**), compared to that of respective control. Three different days of sacrifice indicated by D1, D3, D7 represent day1, day3 and day7. Four different treatment groups with legitimate controls comprising MPTP, Estrogen supplemented MPTP, Tamoxifen supplemented MPTP and Estrogen + Tamoxifen supplemented MPTP represented by −/+ sign. Data are presented as mean ± SEM. (n = 5). Asterisks (*) indicate significant differences (p < 0.05, Student’s t-test) in values for different doses compared to respective controls. The same letter within a, b, c, d; e, f, g, h; i, j, k, l indicates no significant differences and different letters indicate significant differences (p < 0.05, ANOVA) between different treatment groups at the same day of sacrifice in male and female mice brain frontal cortex during treatment condition. Hash sign (#), nsf and dollar sign ($) indicates non significant value with different letters in between treated condition in different groups sacrificed at the same day in male and female mice group (**B**,**D**).

It was found that estrogen level either decreased significantly or remained unchanged in frontal cortex of both male and female mice brains compared to respective controls during MPTP treatment (Fig. 5A,D respectively). However the maximum decrease has been found in female frontal cortex after MPTP treatment. Estrogen supplement has shown the elevated level of estrogen initially, but later that level decreased in frontal cortex of both sexes (Fig. 5A,D). Again, when estrogen receptors are blocked by tamoxifen supplement, the estrogen level decreased in frontal cortex of both sexes after MPTP treatment. Interestingly, estrogen supplement could not increase the estrogen level when estrogen receptors are blocked in MPTP treated frontal cortex of both the sexes (Fig. 5A,D respectively). Blood estrogen level in male and female, variably changed during three different days of sacrifice after MPTP treatment and estrogen supplement either increased the estrogen level in blood or that level remained unchanged in blood plasma compared to the respective control of both the sexes after MPTP treatment (Fig. 5B,E). Most importantly, similar to the brain level of estrogen, when estrogen receptors are blocked, estrogen level in blood plasma decreased significantly compared to their respective control in both the sexes after MPTP treatment (Fig. 5B,E). However the overall increase or decrease of aromatase level did support the variable level of estrogen in frontal cortex of both the sexes after MPTP treatment or during different supplementation, when animals were sacrificed in three different days (Fig. 5C,F).

### MPTP treatment differentially changes the binding of estrogen to its nuclear and cytosolic receptors ERα and ERβ in frontal cortex of both the sexes

**Figure 6:**
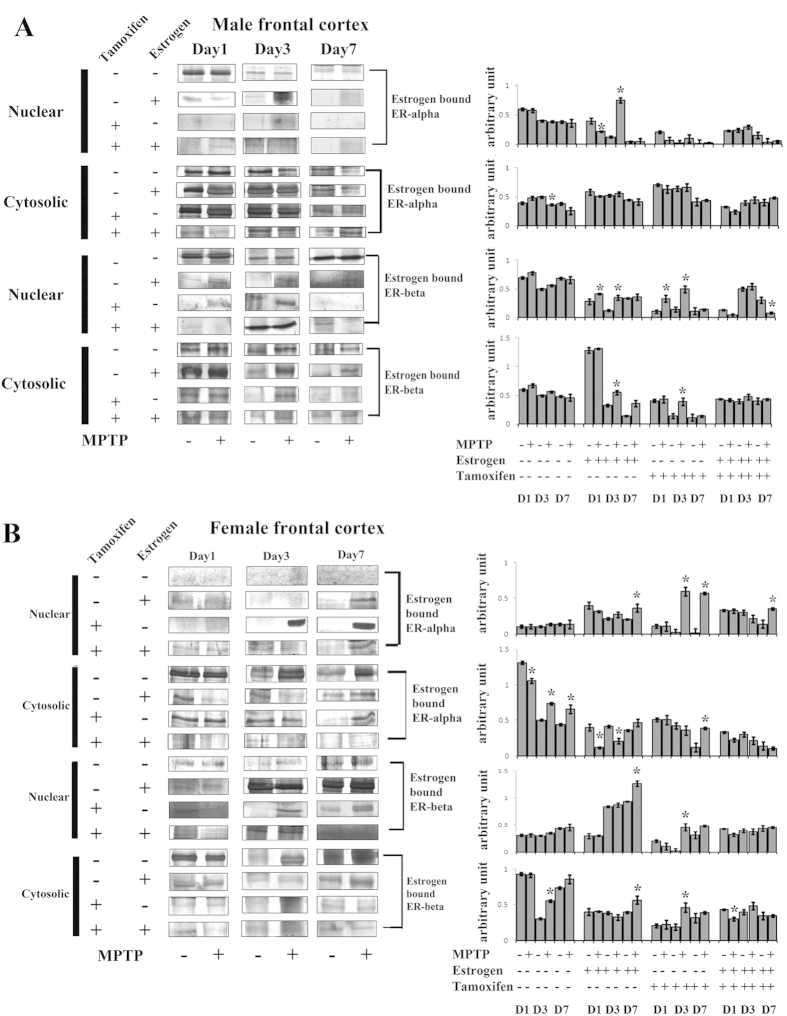
Changes in expression level of estrogen bound estrogen receptor α (ERα) and estrogen receptor β (ERβ) in nuclear and cytosolic fractionation of the frontal cortex of male and female mice brain at three different days of sacrifice in MPTP treatment and also in estrogen and tamoxifen supplemented groups. Immunoprecipitation and analysis of western blots indicate that expression levels of estrogen bound estrogen receptor α (ERα) and estrogen receptor β (ERβ) in nuclear and cytosolic fractionation due to MPTP treatment and also supplemented with estrogen and tamoxifen in male and female frontal cortex (**A,B** respectively). Data are presented as mean ± SEM. (n = 5). Three different days of sacrifice were indicated by Day1, Day3, and Day7 in male and female frontal cortex. Four different treatment groups with legitimate controls comprising MPTP, Estrogen supplemented MPTP, Tamoxifen supplemented MPTP and Estrogen + Tamoxifen supplemented MPTP represented by −/+ sign in respective panels of both male and female frontal cortex (**A,B** respectively). Densitometric analysis has been done by representative bar graphs. Asterisks (*) indicate significant differences (p < 0.05, Student’s t-test) in values for different doses compared to respective controls.

In the present study, in MPTP treated group, the expression level of estrogen bound nuclear estrogen receptor α (nE2ERα) did not alter on all the three days of sacrifice in male (Fig. 6A) but did not express, i.e. estrogen did not bind with ERα at all three days of sacrifice in female frontal cortex (Fig. 6B). Interestingly, in the estrogen supplemented group, the nE2ERα was found to be increased in the middle stage of treated male and in the later stage of treated female frontal cortex (Fig. 6A,B). Notably, when the estrogen receptors blocked, the nE2ERα did not express in male, but expressed in treated female frontal cortex in the middle and the later stages of sacrifice (Fig. 6A,B respectively). Further, in the MPTP treated group, the expression level of estrogen bound cytosolic estrogen receptor α (cE2ERα) increased initially and decreased thereafter in male frontal cortex compared to the control after MPTP treatment (Fig. 6A) however that expression level increased significantly in the later stage of sacrifice in female frontal cortex after MPTP treatment (Fig. 6B). Estrogen supplementation decreased the cE2ERα level in both male and female frontal cortex after MPTP treatment (Fig. 6A,B). Tamoxifen did not produce any significant effect on the expression level of cE2ERα in male or female frontal cortex after MPTP treatment (Fig. 6A,B respectively).

Further, in the MPTP treated group, the expression level of estrogen bound nuclear estrogen receptor β (nE2ERβ) did not change significantly compared to the respective controls of male and female frontal cortex (Fig. 6A,B respectively). The estrogen supplement did not produce any appearance of nE2ERβ in male, but increased the expression level of nE2ERβ in female frontal cortex after MPTP treatment (Fig. 6A,B respectively). Tamoxifen supplement did increase the expression level of nE2ERβ in the middle phase of the male and the middle and last phase of sacrifice in female frontal cortex after MPTP treatment (Fig. 6A,B respectively). In the MPTP treated group, the expression level of cytosolic estrogen bound estrogen receptor β (cE2ERβ) did not change on day1, an increase on day3 and remained same on day7 compared to the controls; however cE2ERβ did not change on day1, increased on day3 and day7 of sacrifice compared to the controls in female frontal cortex of the MPTP group (Fig. 6A,B). The estrogen supplement did increase the expression level of cE2ERβ in male but not in female frontal cortex after MPTP treatment (Fig. 6A,B respectively). Interestingly blocking of estrogen receptors by tamoxifen decreased the expression level of cE2ERβ in both male and female frontal cortex (Fig. 6A,B respectively).

### MPTP treatment differentially change the expression pattern of NF-κB signaling molecules in its nuclear and cytosolic fractionation in frontal cortex of both the sexes

**Figure 7:**
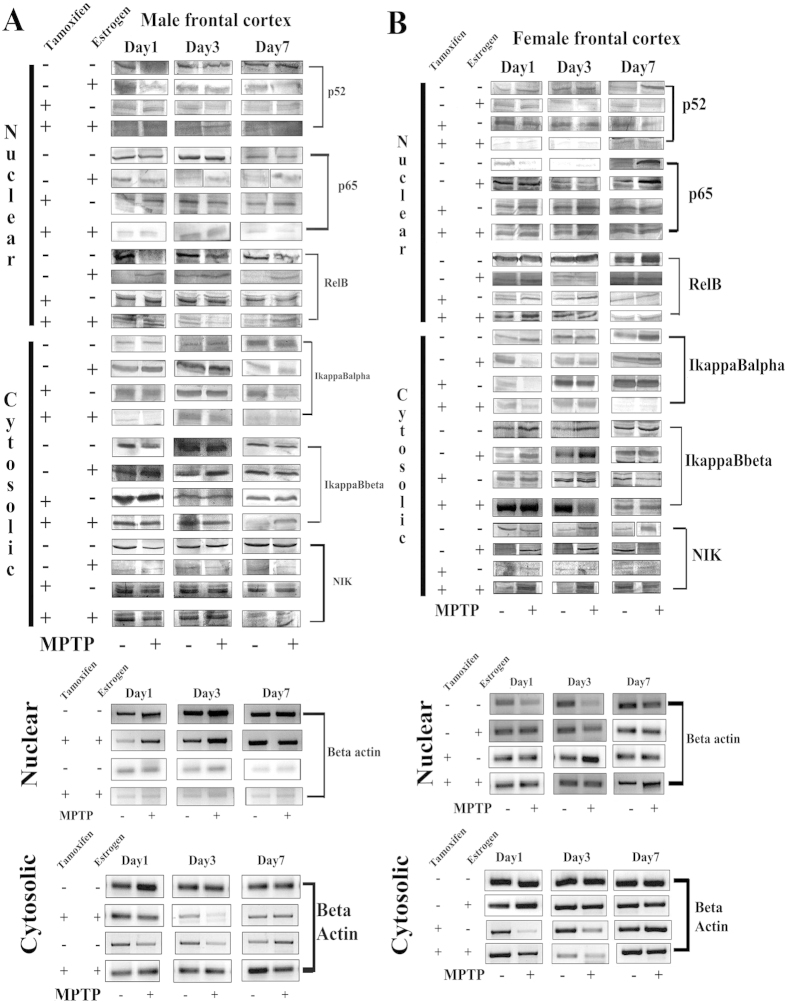
Changes in expression level of p52, p65, RelB, IκBα, IκBβ and NIK in respective nuclear and cytosolic fractionation of the frontal cortex of male and female mouse brain at three different days of sacrifice in MPTP treatment and also in estrogen and tamoxifen supplemented groups. Analysis of western blots indicates that expression levels of p52, p65, RelB, IκBα, IκBβ and NIKin nuclear and cytosolic fractionation in male and female frontal cortex due to MPTP treatment and also supplemented with estrogen and tamoxifen (**A**,**B** respectively). Data are presented as mean ± SEM. (n = 5). Three different days of sacrifice were indicated by Day1, Day3, and Day7 in male and female frontal cortex. Four different treatment groups with legitimate controls comprising MPTP, Estrogen supplemented MPTP, Tamoxifen supplemented MPTP and Estrogen + Tamoxifen supplemented MPTP represented by −/+ sign in respective panels of both male and female frontal cortex (**A**,**B** respectively).

It has been found that in the MPTP treated male group of frontal cortex, the higher expression level and nuclear translocation of p52 was found in all the three days of sacrifice in frontal cortex of both the sexes [(44 ± 3.7)%* p < 0.05] (Fig. 7A,B respectively). However, estrogen supplement decreased that expression level p52 in both the sexes [(34 ± 4.1)%* p < 0.05] after MPTP treatment. Further by tamoxifen supplement, the nuclear translocation of p52 remained same in both female (Fig. 7B) and male frontal cortex (Fig. 7A) after MPTP treatment. It seems to have found that estrogen might have an inhibitory role on p52 translocation into the nucleus (Fig. 7A,B).

The nuclear translocation of p65 increased mostly at a later stage of female [(45 ± 3.7)%* p < 0.05] and middle stage [(29 ± 3.1)%* p < 0.05] of male after MPTP treatment (Fig. 7A,B respectively). Again in the estrogen supplemented group, p65 expression level at a nuclear counterpart of male remained same, but increased only in later stage of sacrifice in female frontal cortex [(59 ± 4.7)%* p < 0.05] after MPTP treatment. Further, blocking of estrogen receptors by tamoxifen promoted the nuclear translocation of p65 in both sexes but increased only at early and middle stage of female [(49 ± 5.1)%* p < 0.05] and decreased in middle stage of male [(37 ± 4.7)%* p < 0.05] frontal cortex after MPTP treatment. In case of p65 nuclear translocation, male and female frontal cortex differs mostly either during estrogen and/or tamoxifen supplementation (Fig. 7A,B).

The nuclear translocation of RelB decreased [(65 ± 5.7)%* p < 0.05] on all three days of sacrifice of MPTP treated male frontal cortex compared to the controls, but in the female, nuclear translocation of RelB became same on day1 and day3 but increased on day7 [(43 ± 4.3)%* p < 0.05] compared to the respective controls during MPTP treatment (Fig. 7A,B). When estrogen supplemented, the RelB expression level on nuclear counterpart of male frontal cortex increased in male [(48 ± 3.7)%* p < 0.05], but remained same in female frontal cortex after MPTP treatment (Fig. 7A,B). When estrogen receptors are blocked, the nuclear translocation of RelB became same compared to the controls in frontal cortex of both the sexes after MPTP treatment (Fig. 7A,B).

The expression level of cytosolic IκBα and IκBβ did support the status of nuclear translocation of p52, p65 and RelB in frontal cortex of both the sexes during MPTP treatment or estrogen supplement and tamoxifen supplement (Fig. 7A,B). The cytosolic expression level of *NIK* decreased on day1 [(24 ± 2.9)%* p < 0.05], remained same on day3 and day7 of the sacrifice of MPTP treated male frontal cortex compared to the controls. In females, *NIK* decreased on day1 [(25 ± 2.7)%* p < 0.05] and increased on day3 and increased on day7 [(47 ± 4.1)%* p < 0.05] compared to the respective controls during MPTP treatment. The *NIK* expression level of estrogen supplemented MPTP treated group was found to be faint on all three days of sacrifice compared to the controls in males; however that expression level increased on day1 and day3 [(39 ± 5.2)%* p < 0.05], decreased on day7 [(53 ± 6.7)%* p < 0.05] compared to their controls in female (Fig. 7A,B). Further, in the tamoxifen treated male frontal cortex, the *NIK* expression level became same on all three days of sacrifice compared to the respective controls, but with the same group of female frontal cortex, the expression level of *NIK* was faint on all three days of sacrifice compared to their respective controls (Fig. 7A,B). Therefore, blocking of estrogen receptors did not have any significant effect on the NIK expression in male frontal cortex, but have a negative effect on female frontal cortex (Fig. 7A,B).

### MPTP treatment differentially change the binding pattern of ERβ with ERα, p65 and RelB in its nuclear fractionation in frontal cortex of both the genders

It has been found that estrogen receptor β deferentially bind with estrogen receptor α (ERβERα complex) forming a heterodimer. However, there is no true evidence has been found whether the estrogen receptors specially the ER-β can directly interact with the NF-κB molecules like p65 (ERβp65 complex) and bind with RelB (ERβRelB) at nuclear fractionation during MPTP treatment or not. In this study, we have put forward the objective to find whether the estrogen in respect with its receptors and NF-κB crosstalk exist in frontal cortex of both the sexes during MPTP mediated PD and neuroinflammation or not.

**Figure 8:**
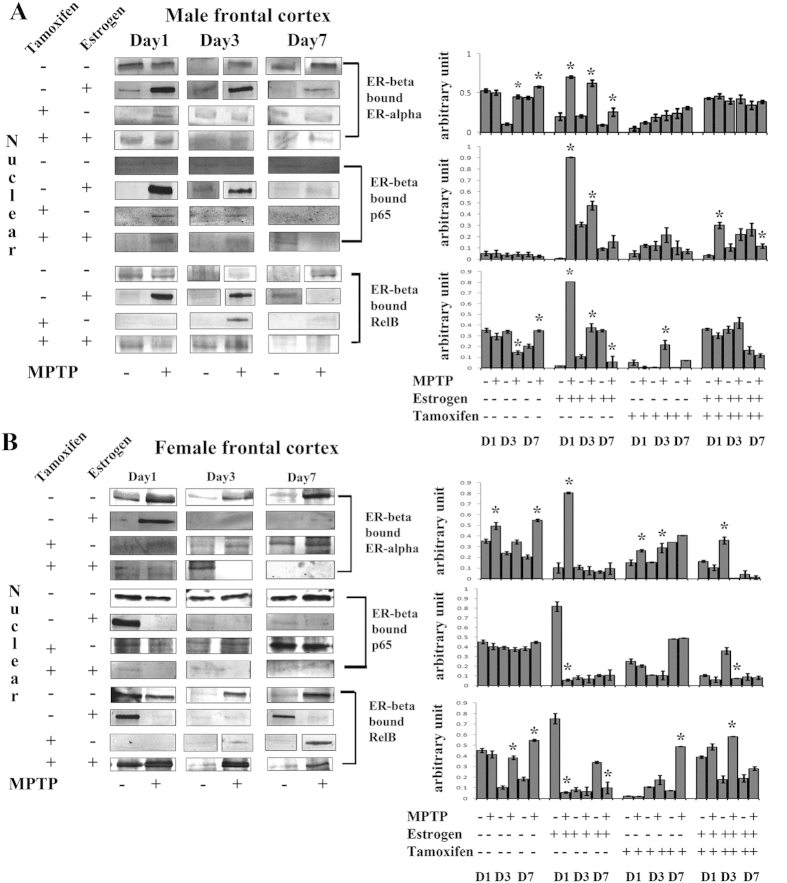
Changes in expression level of estrogen receptor β (ERβ) bound estrogen receptor α (ERα), p65 and RelB in nuclear and cytosolic fractionation of male and female mice brain frontal cortex at three different days of sacrifice in MPTP treatment and also in estrogen and tamoxifen supplemented groups. Immunoprecipitation and analysis of western blots indicate that expression levels of estrogen receptor β (ERβ) bound estrogen receptor α (ERα), p65 and RelB in nuclear fractionation due to MPTP treatment and also supplemented with estrogen and tamoxifen in male and female frontal cortex (**A**,**B** respectively). Data are presented as mean ± SEM. (n = 5). Three different days of sacrifice were indicated by Day1, Day3, and Day7 in male and female frontal cortex. Four different treatment groups with legitimate controls comprising MPTP, Estrogen supplemented MPTP, Tamoxifen supplemented MPTP and Estrogen + Tamoxifen supplemented MPTP represented by −/+ sign in respective panels of both male and female frontal cortex (**A**,**B** respectively). Densitometric analysis has been done by representative bar graphs. Asterisks (*) indicate significant differences (p < 0.05, Student’s t-test) in values for different doses compared to respective controls.

In the MPTP treated group, the expression level of ERβERα became same on day1, increased on day3 and day7 compared to the control; however ERβERα did appear increasingly on all three days of sacrifice compared to the controls in female frontal cortex of the MPTP group (Fig. 8A,B). When estrogen supplemented, in MPTP treated male group, the expression level of ERβERα increased on day1and day3 but faintly appeared on day7; however, in female frontal cortex of the same group, ERβERα did express only on day1 (Fig. 8A,B). In tamoxifen supplemented MPTP treated group, ERβERα appeared faintly on all three days of sacrifice in both male and female frontal cortex (Fig. 8A,B).

In the MPTP treated male group, the expression level of bounded ERβp65 became absent on all days of sacrifice, but that expression level became same on day1 and on day3 and increased on day7 compared to the controls in female frontal cortex (Fig. 8A,B). When estrogen supplemented prior to MPTP treatment, the expression level of ERβp65 appeared on day1 and day3 of sacrifice in male, but did not appear in female frontal cortex compared to the controls (Fig. 8A,B). When tamoxifen was supplemented prior to MPTP treatment, the ERβp65 did not appear on all three days of sacrifice in male and expressed faintly on day1and on day3 and remained same on day7 compared to the control in female frontal cortex (Fig. 8A,B). However, when estrogen supplemented and estrogen receptors are blocked, ERβp65 did not appear in frontal cortex of both the sexes after MPTP treatment (Fig. 8A,B).

In the MPTP treated male group, the expression level of ERβRelB became same on day1, decreased on day3 and increased on day7 compared to the control. However ERβRelB decreased on day1, increased on day3 and on day7 of sacrifice compared to the controls in female frontal cortex of the MPTP group (Fig. 8A,B). When estrogen was supplemented, the expression level of ERβERα appeared on day1 and day3 of sacrifice in male and expressed in all the three days of sacrifice after MPTP treatment in female (Fig. 8A,B). When tamoxifen was supplemented, ERβRelB appeared only on day3 of male and on day7 of female after MPTP treatment compared to the respective controls (Fig. 8A,B). However, in both the estrogen and tamoxifen supplemented group, ERβRelB remained same on day1 and on day3 but did not appear on day7 in treated male frontal cortex and increased on all the three days of sacrifice in female frontal cortex after MPTP treatment (Fig. 8A,B).

## Discussion

The oxidative stress and the mitochondrial dysfunction, preceding DNA fragmentation, could be early events in the apoptotic or necrotic process induced by GSH depletion by GST activation in neuronal cells^19^,^20^ marked by NeuN (neuronal nuclei) which is a neuron-specific nuclear protein and Fox-3 gene product^21^. Astrocytes have neuroprotective role and help to maintain the plasticity and integrity of dopaminergic neurons during MPTP mediated neurodegeneration^22^ and destructive roles in PD path physiology where astrocytes also release proinflammatory cytokines and other toxic molecules linked with the dopaminergic neuronal loss^23^. Increased expression of glial fibrillary acidic protein (GFAP) by ROS, represents astroglial activation and gliosis during neurodegeneration^24^,^25^. Significant evidences report that the microglial cells play a central role in the degeneration of neurons by secreting pro-inflammatory cytokines in response to MPTP mediated oxidative stress in animal models of PD^26^,^27^. To evaluate the status of microglial status, we have used ionized calcium binding adaptor molecule 1 (Iba1) is a microglia/macrophage-specific calcium-binding protein as marker and the Iba1 expression may be associated with microglial activation^28^,^29^. It’s well known that astrocytes convert MPTP to MPP+ ions and render to the neurons^30^. Astrocytes and microglia are the key players in neuroinflammatory responses, by secreting an array of pro- and anti-inflammatory cytokines, anti-oxidant and neurotrophic factors in animal models of PD^31^. In several animal models of PD, it has been found that astrocytes function as neuroprotectant by secreting BDNF, NGF like molecules^18^. However, there is no clear evidence presented about the status of astrocytes during MPTP mediated neurodegeneration in both male and female frontal cortex. In our study, first time we have reported that astrocyte population status and GFAP expression pattern variably changes during the progression of MPTP mediated neurodegeneration in frontal cortex of both the sexes. The decreasing population of neuronal cells took the response from astrocytes. However the activity pattern and population size of astrocytes variably changed according to the sex differences in frontal cortex of both the sexes. The results indicate that the population size or number of astrocytes reacts variably according to the estrogen level and functionality of estrogen via its receptors during the progression of neurodegeneration. However, previous studies only indicated that estrogen function via ER-α in astrocytes promote neuroprotection but not via ER-β during neurodegeneration^32^,^33^. The interlinking of microglial and astrocytic association is a clear evidence of inflammatory response associated with neuronal cell loss. But the exact role of this association still remains elusive^34^. Microglial reactivity not only depends on the neurodegeneration but also on the neurotoxin used and the brain regional heterogeneity^35^. Microglial population size and activation is also determined by the level of estrogen and functionality of estrogen via its receptors. However, that microglial activation and population varied as per sex differences, progression stages of neurodegeneration and on the ROS level during MPTP treated mouse model of PD. Previous studies indicated that estrogen function has a positive role in attenuating the reactive gliosis^36^. Proinflammatory cytokines like TNF-α carry out the progressive neural loss by neuroinflammation^37^. TNF-α derived from activated microglia also function as an autocrine mediator in microglial activation^38^. Therefore, to evaluate the neuroinflammatory status in the brain, we have chosen the proinflammatory cytokine TNF-α as a marker in our study. The activation pattern of astrocytes and microglial cells has revealed that not only the endogenous supply (by glial cells) but also a peripheral supply of TNF-α was present in different days of sacrifice in male and female frontal cortex during MPTP treatment. However, estrogen has a positive role in decreasing the TNF-α level in frontal cortex of both the sexes after MPTP treatment and the finding supports the evidence for the involvement of microglia in neuroinflammation and the anti-inflammatory activity played by estrogens specifically in microglia^39^. Alternatively the neuroprotective molecule estrogen level decreased mostly in frontal cortex of both the sexes. The decrease of estrogen either because of less activation of astrocytes that synthesizes estrogen during the pathogenic condition^40^,^41^ or the synthesizing enzyme aromatase level decreased in frontal cortex of both the sexes during MPTP treatment. It has been found that the peripheral increase of supply in estrogen did not support the brain level of estrogen all the time in both the sexes after MPTP treatment.

The sole presence of estrogen or the activity of estrogen via binding to its receptors in both the nuclear or cytosolic part, have implication in neuroprotection in MPTP treated mouse model of PD^42^. In the absence of nuclear or cytosolic estrogen receptors, the neuroprotective action of estrogen becomes sluggish or less effective^43^. The function of ER-α in neuroprotection is well established^44^ but the role of ER-β is not well established still now. The binding of estrogen with its receptors in nuclear and cytosolic counterpart varied according to sex specific or brain region specific during PD condition. That binding may also depends on level of endogenous estrogen in brain in particular disease condition. However, there is no documentation has been made still now what the binding status of estrogen with its nuclear and cytosolic counterpart in frontal cortex of both the sexes during the progression of PD. Our study for the first time evidenced the binding status of estrogen with its receptors during MPTP mediated progressive neurodegeneration.

When neuroinflammation persist during the progression of the disease in different days of sacrifice, we have found that selected NF-κB molecules expression pattern changes differently in frontal cortex of both the sexes. It has been reported that neuronal loss may be caused due to increased cytokines and apoptosis-related proteins via the activation of NF-κB in reactive astrocytes of the substantia nigra after MPTP treatment in mice^45^ and the NF-*κ*B as a Target for Therapy in Parkinson’s Disease has already been revealed previously^46^.

However which pathway (canonical or non canonical pathway) persists during MPTP mediated neurodegeneration, is still remaining unknown. The pattern of expression level of NF-κB molecules like p65, p52, RelB and NIK changed according to the changes in estrogen level and functionality of estrogen via its nuclear and cytosolic receptors counterpart in frontal cortex of both the sexes. There is no true evidences have been found still now that how NF-κB pathways modulate during the progression of PD in frontal cortex of both the sexes and neuroinflammation persist. In our study, we have found that during the progression of the neurodegeneration in MPTP model, both the canonical or non canonical pathway exist alternatively in male and female mouse brain frontal cortex.

As previously mentioned that estrogen has anti-inflammatory property that may/may not be actionable by estrogen receptors in the present study, we also examined whether there persist any direct interaction between estrogen receptor β and NF-κB molecule p65 or RelB. There is evidence that estrogen can activate the NF-κB pathway via nongenomic pathways and increase cellular resistance to injury^47^. However, there is no documentation yet whether the same phenomenon exists in MPTP mediated neurodegeneration in the frontal cortex or not. Previous report exists for inhibitory interaction between NF-κB and estrogen receptor in cancer and during pregnancy^48^ but no report has been found in the frontal cortex of male and female mouse brain during MPTP treatment. Previously, mentioned that estrogen inhibits p65 intracellular transport to the nucleus. This activity is selectively mediated by estrogen receptor α (ERα) and not ERβ^49^. But the sparse presence of estrogen bound ER-β in frontal cortex lead us to investigate the possible role of ER-β during the MPTP mediated neurodegeneration in frontal cortex of both the sexes; whether ER-β do interact with ER-α as a heterodimer and bind with NF-κB molecule p65 or RelB during the neurodegeneration or not. It has been found that binding of heterodimeric or homodimeric nuclear ER-β with NF-κB molecules variably changed during the progression of the MPTP mediated neurodegeneration. That might implicate an important role of nuclear ER-β during NF-κB mediated neuroinflammation in frontal cortex of both the sexes. However, as neuroinflammation via TNF-α still proceeds in the MPTP treated brain therefore it may be assumed that the binding of NF-κB molecules with homodimeric or heterodimeric nuclear ER-β either don’t have any negative effect on neuroinflammation or it may promote the neuroinflammation.

The level of neuroinflammatory TNF-α in the brain depends on the activation pattern of glial cells (both microglia and astrocytes) in both the sexes in different days of sacrifice after MPTP treatment and that level of TNF-α depends on the decreased level of estrogen and functionality of estrogen by its receptors. Nuclear and cytosolic estrogen bound ER-α and ER-β differently changed during MPTP treatment in both the sexes and that binding with its expression level may be dependant on the functional status of estrogen receptors. However, no positive correlation has been established in the present study. Both the canonical and non canonical pathways exist during the MPTP mediated progression of the disease. And there is a positive binding of ER-β exists with the NF-κB molecule p65 and RelB but that could not able to suppress the NF-κB mediated neuroinflammation during the progression of the disease mediated by MPTP.

These vast and rapid changes in molecular and cellular pattern during the course of the MPTP intoxication ultimately promote neuroinflammation that leads to neurodegeneration. Both the canonical and/or non canonical pathways of NF-κB exist in frontal cortex of both the sexes after MPTP treatment that is why neuroinflammation still progress. The homodimeric or heterodimeric form of ER-β binds with NF-κB molecules p65 and RelB differently, but the canonical or non canonical pathways of NF-κB molecules could not be stopped or may be promoted and neuroinflammation proceeds. One positive correlation is that the changes in the molecular and cellular pattern in frontal cortex of both the sexes during MPTP mediated neurodegeneration depend on the functionality of estrogen with its nuclear or cytosolic estrogen receptors α and β. In addition, the present experiments reinforce further studies to unveil the physiological and pathophysiological significances of the cellular and molecular interactions in different regions of mammalian brain and their sex differences during healthy and disease conditions. Therefore the diversified variability of estrogen level and its action via both the receptors have a positive implication that male is more prone to PD risk than female.

## Methods

### Animal and treatment

Swiss albino male and female mice (~25 g each; five mice/group) obtained from the National Institute of Nutrition (Hyderabad, India) were housed in a departmental animal facility (located at the Animal Housing Unit in the Department of Zoology, University of Calcutta; maintained at 25–28 [±2] °C; with 55 [±5] % relative humidity, and a 12 hr/12 hr light/dark cycle). Animals were provided rodent chow (National Institute of Nutrition) and filtered water *ad libitum.* All animal experiments were performed following the “Principles of Laboratory Animal Care” (NIH publication No. 85-23, revised 1985), and following the specific Indian law on “Protection of Animals” under the supervision of authorized investigator and the methods were carried out in accordance with the approved guidelines of the Institutional Animal Ethics Committee (IAEC). The experimental protocols were also approved by the IAEC at the Department of Zoology, University of Calcutta.

The experimental mice of both sexes (n = 5) were randomly divided into different groups comprising (A) vehicle (saline) treated control (B) MPTP-treated: Animals received four subcutaneous injections of MPTP [18 mg/kg B.w., Sigma Aldrich, Inc. (St. Louis, MO)] at 2 hours interval in a single day and were sacrificed at 24 hours (day1), day3 and day7 after the last dose was administered (53), (C) 17-β estradiol-treated: Animals were administered 17-βestradiol (2μg/kg b.w., Sigma Aldrich, Inc. [St. Louis, MO)] intraperitoneally once a day for 5 consecutive days and was sacrificed on the day2, day4 and day8 after the last dose was administered, (D) Tamoxifen (Estrogen receptor antagonist)-treated: Animals received intraperitoneal injections of tamoxifen [5mg/kg b.w., Sigma Aldrich, Inc. (St. Louis, MO)] once a day for 5 days and was sacrificed on the day2, day4 and day8 after the last dose of tamoxifen administered., (E) 17-βestradiol + MPTP treated: Animals administered 17-β estradiol (2 μg/kg b.w.) intraperitoneally once a day for 5 days prior to MPTP treatment and was sacrificed on day1, day3 and day7 after MPTP treatment (day0). (F) Tamoxifen + MPTP treated: The tamoxifen + MPTP treated set received intraperitoneal injections of tamoxifen (5 mg/kg b.w.) once a day 5 days prior to MPTP treatment day and was sacrificed on day1, day3 and day7 after MPTP administration (day0). (G) 17-βestradiol + Tamoxifen treated: Animals received Tamoxifen treatment first (as mentioned above) and after four hours, received 17β estradiol (as mentioned above) for five consecutive days and sacrificed at day2, day4 and day8 after receiving the last dose and (H) 17-βestradiol + Tamoxifen + MPTP treated: Animals received Tamoxifen treatment first (as mentioned above) and after four hours, received 17β estradiol for five consecutive days and sacrificed at day1, day3 and day7 after MPTP treatment (day0). At 24 hrs, 72 hr and day7 after the final dosing, the mice in each group were euthanized by an overdose with sodium thiopentone (Mancure Drugs Private Ltd., Mumbai, India) and brain tissue were then harvested for analyses as described in the various assays below.

For estrous cycle, we placed the tip of plastic pipette, filled with PBS or saline (~10 μL), into the vagina. Then we flushed the vagina gently three to five times with same PBS/saline solution. Then we collected final flush with a pipette tip. A volume of 10 μL of saline solution allows collecting sufficient material for observation of vaginal cytology. Then we placed the final flush containing vaginal fluid on a glass slide. Then we observed unstained material under light microscope with a 10× objective. To observe the estrus stage, we identified cornified squamous epithelial cells in clusters, no visible nucleus with a granular cytoplasm and the irregular shape. The vaginal smear was taken thrice daily sparing eight hours for each female animal for the whole treatment period up to sacrifice point.

### Immunohistochemistry

Immunohistochemistry was performed according to Mitra *et al.*, 2011^50^; briefly, after the decapitation of euthanized animals, brains were removed immediately and washed in ice cold phosphate-buffered saline (PBS, pH 7.4). Sagittal brain sections (5 μm thick) were cut from paraffin-embedded brain tissue and mounted on positively charged Super frost slides (Export Mengel CF, Menzel, Braunschweig, Germany). Tissues were deparaffinized, dehydrated through graded alcohols, and peroxides quenching was done in 3% hydrogen peroxide solution. Background staining was inhibited with 5% bovine serum albumin [BSA] (Sisco Research Laboratories Pvt. Ltd. [SRL], Mumbai, India) and then incubated in a humid chamber overnight at 4 °C with primary antibodies (diluted 1:50 in 5% BSA). Primary antibodies like- anti-FOX3 (mouse monoclonal), and anti-GFAP (mouse monoclonal) Abcam plc (Cambridge, UK) used in separate cases. After washing in PBS-Tween20, sections were sequentially incubated with horseradish peroxidase (HRP) -conjugated anti-sera specific for those antigens at 1:30 ratio in Tris-buffered saline containing 0.3% Triton-X and 0.5% blocking agent for 2 hours at room temperature. Immunoreactive complexes detected by using a DAB system of Bangalore GeNei Pvt. Ltd. (Bangalore, India). Sections were counterstained briefly with hematoxylin, dehydrated through graded alcohols, cleared in xylenes, and cover slipped with the DPX mounting medium. Slides that received no primary antibody served as negative controls. Images were captured using a U-TVO 63× C microscope (Olympus Corp., Tokyo, Japan) having 40X lens.

### Preparation of total cell homogenate

Total cell lysate for western blot analysis were performed according to Mitra *et al.*, 2011^50^. Briefly, FC– was dissected out immediately after the decapitation according to the Paxinos Mouse Brain Anatomy Atlas. Tissues were homogenized in ice-cold RIPA lysis buffer (150 mM sodium chloride, 1.0% TritonX-100, 50 mM Tris, pH 8.0, 0.01% SDS, 0.5% sodium deoxycholate) containing 1 mM PMSF (phenyl methane sulfonyl fluoride or phenylmethylsulfonyl fluoride) (SRL, India), 1 μg/ml each aprotinin, leupeptin and pepstatin (Sigma-Aldrich Inc., USA). The samples were sonicated and incubated on ice for 30 min, and centrifuged 3 times at 14,000 RPM for 15 min at 4 °C. A portion of the supernatant was reserved for protein determination using the Bradford reagent (Sigma-Aldrich Inc., USA) and subsequent measurement of absorbance was done at 595 nm in a UV-1700 Pharma Spec, Shimadzu spectrophotometer (Shimadzu Scientific Instruments, Columbia, MD).

### Cytosolic and Nuclear Extract Preparation

According to Arumugam *et al.*, 2011^51^, mouse brain tissue pellets were suspended in 1.5 ml of buffer A (10 mM Hepes, pH 7.9, 1.5 mM MgCl2, 10 mM KCl, and 1 mM EDTA) supplemented with 1 mM dithiothreitol, 1 mM phenylmethylsulfonyl fluoride, 0.5 μg/ml leupeptin, 1 μM pepstatin, and 0.05% Nonidet P-40, and left on ice for 10 min. The nuclei were separated from the cytosolic fraction by centrifugation at 4 °C at 3000 RPM for 10 min. Supernatants were collected as cytosolic protein extracts. To prepare nuclear extracts, the pellets were resuspended in 1.2 ml of buffer B (20 mM Hepes, pH 7.9, 1.5 mM MgCl_2_, 420 mM KCl, 25% glycerol, and 0.2 mM EDTA) supplemented with 1 mM dithiothreitol, 1 mM phenylmethylsulfonyl fluoride, 0.5 μg/ml leupeptin, and 1 μM pepstatin, homogenized and left on ice for 30 min. Samples were centrifuged at 14,000 rpm for 20 min at 4 °C. The supernatant, containing nuclear proteins, was separated, used in a Bradford assay and the remainder was stored at −80 °C for further experiments.

### Western blot analysis

According to Mitra *et al.* 2011^50^, 40 micrograms of total protein extracts, cytosolic and nuclear protein extracts of each sample of each day of sacrifice were separated on a 9–12% polyacrylamide gels, electro blotted onto polyvinylidene difluoride membrane (Amersham Biosciences, Piscataway, NJ), and the membranes were blocked with 5% nonfat dry milk for 1-hr., then incubated with primary antibodies, anti-Iba1, anti-ERα, anti-ERβ, RelB, p65, p52, IκBβ, IκBα, NIK [Abcam plc. (Cambridge, UK)], overnight at 4 °C, washed in TBS-Tween-20 (0.01%) and then incubated with AP-conjugated secondary antibodies at a dilution of 1:2000 for 2-hrs at room temperature. The membranes were then developed with NBT/BCIP (nitroblue tetrazolium chloride/5-Bromo-4-chloro-3-indolyl-phosphate; Hi-Media, Mumbai, India) and the band intensity was measured by densitometry (Gel Doc™ XR+ System, BioRad Laboratories, USA) following β-Actin as loading control.

### Immunoprecipitation

Immunoprecipitation was done according to Kundu *et al.*, 2009^52^. Briefly, about 100 μg of protein from the cortical list of of both male and female mice were immunoprecipitated using 10 μl each of anti-estrogen, anti-ERβ separately [Abcam plc (Cambridge, UK)] for overnight at 4 °C with gentle rotation. 25 μl Protein G CL-Agarose (Bangalore Genei, India) was added to the previous mixture, depending on the experiment and allowed it to mix for 4 hours at 4 °C with gentle rotation. It was then centrifuged at 3000 RPM for 2 min. The immunoprecipitates were washed extensively with sterile PBS and separated by SDS-PAGE, followed by western analyses with anti-ERα, anti-ERβ, RelB, p65, antibody in separate cases as described above.

### ELISA

Dissected brain region homogenized in specialized lysis buffer, containing 25 mM HEPES, 0.1% Tween-20, 5 mM magnesium chloride, 1.3 mM EDTA, 1 mM EGTA and protease inhibitors (1 mM phenylmethylsulfonyl fluoride, 0.5 μg/ml leupeptin, 1 μM pepstatin). Anti-estrogen, anti-aromatse and anti-TNFα mouse monoclonal antibodies [Abcam plc (Cambridge, UK)] used to detect the level of the respective molecules in brain lysate and in blood plasma. The Estrogen and TNF-α level in FC lysates were measured using commercial enzyme-linked immunosorbent assay (ELISA) kits according to the manufacturer’s protocol (R&D Systems, Minneapolis, MN, USA). The OD values were read in a microplate reader at 450 nm. The concentrations of TNF-α and estrogen in the sample were calculated against the standard curve generated using recombinant TNF-α.

### FACS analysis of percentage of neuronal and glial cell population

Single cell suspension from FC of mice were incubated with anti-CD16/32 (FC-block), followed by fixation with 4% paraformaldehyde and permeabilization with 0.1% saponin. The cells were then fixed, washed twice with cell staining buffer and intracytoplasmic staining was performed using anti-FOX3 for neuronal cells, anti-Iba1for microglial cells, and anti-GFAP for astrocytic cells in separate vials and incubated for 4 hours at room temperature. Rabbit polyclonal to anti-goat secondary antibody and Alexa-flour488 conjugated anti-mouse and APC conjugated anti-mouse secondary antibodies [Abcam plc (Cambridge, UK)], FITC-conjugated secondary mouse anti-goat antibody [Santa Cruz biotechnologies (DA, USA)] were added to the respective primary antibody containing vial and incubated for 2 hours at room temperature. The percentage of respective cell types were then analyzed in a FACS Aria III with FACS diva software (BD bioscience). Viable cells were gated by forward and side scattering. Representative bar graphs indicated as percentage of cells.

### Statistical analysis

Values between groups were analyzed using single way ANOVA and two way ANOVA where appropriated. All values are shown as mean ± SEM, except where otherwise indicated. Data were analyzed and when appropriate, the significance of the differences between mean values was determined using Student’s t-test. The results were considered significant at p < 0.05.

## Additional Information

**How to cite this article**: Mitra, S. *et al.* Alteration in Nuclear Factor-KappaB Pathway and Functionality of Estrogen via Receptors Promote Neuroinflammation in Frontal Cortex after 1-Methyl-4-Phenyl-1,2,3,6-Tetrahydropyridine Treatment. *Sci. Rep.* **5**, 13949; doi: 10.1038/srep13949 (2015).
